# Using Short Dietary Questions to Develop Indicators of Dietary Behaviour for Use in Surveys Exploring Attitudinal and/or Behavioural Aspects of Dietary Choices

**DOI:** 10.3390/nu7085287

**Published:** 2015-08-04

**Authors:** Alison Daly, Christina M. Pollard, Deborah A. Kerr, Colin W. Binns, Michael Phillips

**Affiliations:** 1 School of Public Health, Curtin University, Kent Street, Perth 6845, Australia; E-Mails: D.Kerr@curtin.edu.au (D.A.K.); C.Binns@curtin.edu.au (C.W.B.); 2 Department of Health in Western Australia, 189 Royal Street, East Perth 6004, Australia; E-Mail: Christina.Pollard@health.wa.gov.au; 3 Harry Perkins Institute for Medical Research, University of Western Australia, 50 Murray Street, Perth 6000, Australia; E-Mail: michael.phillips@perkins.uwa.edu.au

**Keywords:** dietary behaviours, healthful eating indicators, structural equation modelling, cross sectional

## Abstract

For countries where nutrition surveys are infrequent, there is a need to have some measure of healthful eating to plan and evaluate interventions. This study shows how it is possible to develop healthful eating indicators based on dietary guidelines from a cross sectional population survey. Adults 18 to 64 years answered questions about the type and amount of foods eaten the previous day, including fruit, vegetables, cereals, dairy, fish or meat and fluids. Scores were based on serves and types of food according to an established method. Factor analysis indicated two factors, confirmed by structural equation modeling: a recommended food healthful eating indicator (RF_HEI) and a discretionary food healthful eating indicator (DF_HEI). Both yield mean scores similar to an established dietary index validated against nutrient intake. Significant associations for the RF_HEI were education, income, ability to save, and attitude toward diet; and for the DF_HEI, gender, not living alone, living in a socially disadvantaged area, and attitude toward diet. The results confirm that short dietary questions can be used to develop healthful eating indicators against dietary recommendations. This will enable the exploration of dietary behaviours for “at risk” groups, such as those with excess weight, leading to more relevant interventions for populations.

## 1. Introduction

Evidence is increasing that the need to eat well as early as possible is inextricably linked to attainment and maintenance of a healthy weight and overall good health [1,2,3,4]. In 2011–2102, Australia conducted its third national nutrition survey which coincided with the release of the updated Dietary Guidelines for Australia (DGA) in 2013 [5]. The first release of results from the national nutrition survey indicate that the majority of people are not eating a diet consistent with the Dietary Guidelines [6]. Previous reviews have shown that influencing people to eat well is a complex and difficult process [7,8] and that knowledge and attitudes in line with healthy eating do not necessarily translate into behaviour [9]. Many studies have provided important information about aspects of attitudes, beliefs, and behaviours surrounding good eating habits in relation to families [10,11]; socio demographics [12]; predictors of disordered eating behaviours and diet [13], and attitudes towards appearance and diet [14]. One of the difficulties in being able to conduct these necessary investigations in countries where dietary surveys are infrequent, such as Australia, is that there is not enough current information about eating choices. What is needed is an interim measure that captures important aspects of diet that can be used to investigate how people make decisions about what they eat. A recent study showed that it is possible to get an indicator of healthy eating choices using four items [15] and this study is an important step in developing measures that can be used with contextual data to provide a better picture of what drives eating choices. However such measures are limited as they cannot identify areas of diet which may be more important than others in determining problems related to overeating and poor nutrition. The study investigates whether or not it is possible to use the dietary information collected by the Nutrition Monitoring Survey Series (NMSS) to develop a measure of who is meeting dietary guidelines. The Western Australian Department of Health’s NMSS commenced in July/August 1995 to provide information to assist planning interventions promoting the Australian guidelines for healthy eating. The information obtained in these surveys ranges from what people think are problems, how they see their own behaviour, skill or appearance in relation to nutrition, and what they know, believe, and do about the key components of a healthy diet, as defined by the DGA. The surveys are unique in that they collect some food consumption information, as well as knowledge, attitudes, and beliefs that accompany that behaviour. The food consumption part of the NMSS uses short dietary questions to measure consumption of key food groups [16] that have been evaluated against weighed dietary records [17,18]. The questions are used to monitor high level population based adherence to the DGA. These questions are not a measure of dietary intake nor are they a measure of nutrients; rather they are indicators of consumption of selected foods taken from the major food groups recommended for daily consumption. The underlying premise in using these questions to develop a healthful eating indicator is that it can be viewed as a latent indicator of diet quality. If the population is eating recommended serves and types of foods based on dietary guidelines, then they, by definition, must be eating a reasonable quality of diet. While imperfect, this latent assessment of diet quality can be used as a benchmark against which to assess the dietary behaviours and choices at a population level when included in surveys investigating determinants and precursors of diet. This objective of this study was to demonstrate that, with relatively few questions, a robust indicator of eating behaviour can be developed for inclusion in large-scale cross sectional surveys. These indicators have the potential to identify and add context to dietary beliefs, attitudes, and behaviours at a population level.

## 2. Experimental Section

Since 1995, about every three years, over one thousand adults aged 18 to 64 years are interviewed using Computer Assisted Telephone Interviews (CATI) and asked questions about their attitudes and beliefs about diet. The surveys are managed by the Department of Health, who grant ethics approval for the data collection Only the NMSS 2012 survey data were used to develop the healthful eating indicator as it was the most recent survey which contained dietary information across all areas of the DGA. The sample was a stratified random sample according to area of residence drawn from the most recent Electronic White Pages for Western Australia. All sample households with an address were sent a Primary Approach Letter and every household in the initial sample was called up to ten times to achieve contact. Contacted numbers were eliminated if they were not a household or if there was no person living in the household within the age range. Households with more than one adult fulfilling the requirements were asked which adult had the most recent birthday and that adult was selected for interview. No substitutes were permitted. At least ten call backs were made to achieve an interview. Interviews took place during the four weeks between mid-July and mid-August. A raw response rate of not less than 70% was required based on households contacted within the eligible age range whether or not an interview was achieved. In 2012, 1548 people, 1005 females and 543 males, aged between 18 and 64 years, were interviewed, with a response rate of 82.4% based on interviews attained divided by eligible households contacted.

### 2.1. Diet Questions

The NMSS collects information on the previous day’s consumption of food groups identified by the DGA. The food groups covered include vegetable, fruit, cereals, dairy, and fish or meat. Information on fluids used are also collected. The data is self-reported and questions were about the amount and types of foods eaten the previous day. Each question contains a definition of a serve or asks for amounts in common household measures such as cups or spoons, which can be used to convert the amount to serves as defined by the DGA.

### 2.2. Sociodemographic Indicators

Indicators of sociodemographic status included sex, age, education, income, employment status, living arrangements, perceived spending power, and an area-based indication of relative socioeconomic disadvantage known as Socio-Economic Indexes for Areas (SEIFA) and developed by the Australian Bureas of Statistics [19].

### 2.3. Developing the Dietary Guideline Indicator

There are only two dietary indices that have been developed for Australia. Both were based on the 1995 National Nutrition Survey and both used a combination of the frequency foods were eaten; some consumption questions, for example fruit and vegetable consumption; and some behaviours such as whether or not meat was trimmed of fat. The first index, developed in 2007, used a relatively simple construction and had six dimensions based on the 2003 Dietary Guidelines for Australian [20]. The second index, developed in 2008, used a similar conceptual framework but had eleven components exploring more parts of the 2003 Australian Dietary Guidelines which included a measure of alcohol consumption [21]. While the NMSS does not collect information about alcohol consumption, there were more possible comparative scales with the 2008 index than with the 2007 index and for this reason it was selected as the model for the development of a NMSS healthful eating indicator (NMSS_HEI). The NMSS_HEI is based solely on consumption of key food groups the previous day. The dietary guideline index developed in 2008 (DGI_2008) used frequency as a rough indication for amount, with each frequency of consumption assumed to be at least one serve. As the NMSS collects dietary data in amounts they can be converted into serves based on the recommendations for adults aged between 18 and 64 years [22]. To accommodate the differences between frequency and consumption, and to compensate for questions used in the DGI_2008 which were not asked in the NMSS, comparable measures for the NMSS data were developed. For example, in the DGI_2008 saturated fat consumption was based on the type of milk used and whether or not meat was trimmed of fat, but the question about trimming fat from meat was not asked in the NMSS, so saturated fat consumption is made up of the type of milk, cheese, and yoghurt consumed and whether sausages and biscuits (high in saturated fat) were eaten. For type of grains, the DGI_2008 used only whole grain bread, but as there was information available for type of bread, rice, pasta, and breakfast cereals, all were used in scoring the type of grains consumption. The DGI_2008 used lean meat, fish, eggs, nuts and seeds, and legumes/beans as major sources of protein, but the only comparable measure in the NMSS were serves of meat or fish eaten the previous day. Additional foods were also differently assessed. For the NMSS_HEI when people consumed more than the recommended number of serves of a particular food group, the full score was given on the specific food component (e.g., cereals) but any serves above the recommended amount were assessed against the additional serve recommendations for each food group by age and sex [5] and scores based on compliance with these. The only exceptions to the additional food score assessments were fruit and vegetables, as the evidence base indicates that there are no known detrimental effects of consuming more than the recommended amounts of these foods [5,23]. A full description of the way in which the index was constructed is shown in Table 1. The table shows the 2013 ADG recommendation for each part of the scale with the way in which the score was assigned, what constitutes not meeting the recommendation and how derivation of the score differs from the DGI_2008.

**Table 1:** Construction of the NMSS_HEI scale based the 2013 ADG [6] with comparison to DGI_2008 [21], NMSS 2012.

Australian Dietary Guidelines 2013 Using Data Collected in the NMSS 2012	Indication and Description ^a,b^	Criteria for Maximum Score (10)	Criteria for Minimum Score (0)	Difference with DGI _2008 ^c^
Enjoy a wide variety of nutritious foods	The number of different types of core foods eaten on the previous day. The following made up the variety score: vegetables; fruit; dairy and cereals	Eats four types of vegetables (4 was the median); any fruit; consumes one of milk, yoghurt or cheese; eats three types of cereal foods( breads, bread substitutes, breakfast cereals, rice or pasta)	Eats none of the foods	Used proportion of foods for each food group eaten at least once a week
Enjoy plenty of vegetables, including different types and colours, and legumes/beans	Serves of vegetables usually eaten. This question did not specify “yesterday”	For men aged 19–50,at least six serves; for all others at least 5 serves	Eats none	Serves of vegetables & legumes per day
Enjoy fruit	Serves of fruit eaten yesterday	All groups, at least 2 serves	Eats none	Serves of fruit eaten per day
Enjoy grain (cereal) foods	Serves of cereals eaten yesterday	Men & women aged 18, at least 7 serves; men aged 19–64, at least 6 serves; women aged 19–50, at least 6 serves; women aged 51–64, at least 4 serves.	Eats less than recommended	Frequency of consumption
Mostly wholegrain and/or high cereal fibre varieties	Serves of wholegrain or wholemeal cereals eaten yesterday	Full score if all types of cereals eaten yesterday were wholemeal or wholegrain	No cereal foods were wholemeal or wholegrain	Only wholemeal bread was used
Enjoy milk, yoghurt, cheese and/or alternatives, mostly reduced fat ^d^	Serves of dairy foods used/consumed yesterday	Men & women aged 18, at least 3½; men aged 19–64 and women aged 19–50, at least 2½ serves; women aged 51–64, at least 4 serves	Used/consumed no dairy foods yesterday	Frequency of consumption of dairy foods per day
Enjoy lean meats and poultry, fish, eggs, tofu, nuts and seeds, and legumes/beans	Serves of meat or fish eaten yesterday ^e^	Men & women aged 18, at least 2½ serves; Men aged 19–50, 3 or more serves; Women aged 19–50, 2 ½ or more serves; women aged 51–64, 2 or more serves.	Eats less than recommended	Frequency of consumption of meats and alternatives the previous day with proportion of lean.
Limit intake of foods high in saturated fat	Ate full fat dairy food or sausages or biscuits	The numbers of foods eaten were converted to a score out of ten and those who ate none got a score of 10	Ate all foods high in saturated fats	Used type of milk usually consumed as well as trimming fat from meat.
Drink plenty of water ^f^	Litres of fluids - proportion of water to total fluids set at 66% ^d^	Drank at least 8 (250) mL, cups (women) or 10 (250) mL, cups (men) of any fluid yesterday	Drank less than suggested	Used 8 cups (250 mL)
Limit intake of foods and drinks containing added sugars	Number of foods high in added sugar consumed yesterday including biscuits, soft drinks, crumpets, scones, muffins (cake type) and sugary breakfast cereals	No such foods eaten yesterday	Ate three types yesterday	Used frequency of consumption of cordial, fruit juice, soft drinks, jam, chocolate or confectionary
To achieve and maintain a healthy weight, be physically active and choose amounts of nutritious food and drinks to meet your energy needs ^g^	Extra serves of any foods except fruit and vegetables consumed which were above the additional serves guidelines	No additional serves eaten	Any additional serves above upper limit	Used a combination of added sugar and extra foods.

^a^ Serves are estimated using the 2013 ADG definitions; ^b^ The maximum recommended serves or more is the basis for the maximum score but additional serves over recommended and more than recommended additional are then penalised under the extra serves score; ^c^ DGI_2008 DQI used each frequency of consumption to be a rough measure of a serve; ^d^ Dairy foods were weighted by fat content; ^e^ The only available questions on protein were about serves of meat and fish; ^f^ Used the cut points for fluids suggested in Educators guide for the Australian Dietary Guidelines 2013—the reference also suggests that “most” be in the form of water so 66% water was taken as an measure of “most” as there was no quantified amount suggested [24] (National Health and Medical Research Council, 2013); ^g^ The 2013 ADG provides an additional serves guideline for taller and more active adults and this was used to assess extra serves over and above these plus recommended.

### 2.4 Analysis

The total NMSS_HEI was the sum of the eleven individual components of the indicators described in Table 1. As with the previously developed DGI_2008, scores for each component are out of ten and as there are eleven measures, the total possible score is 110, with higher scores indicating the healthier eating. Exploratory factor analysis with confirmatory structural equation modelling (SEM) was conducted on the total NMSS_HEI to best identify the structure of the model [25]. The confirmatory SEM was conducted with the data unweighted, allowing for an estimate of comparative fit [26,27] and then the fit compared a SEM using the data weighted for the survey sample design [28]. Post estimation tests conducted on the structural equation model included the comparative fit index, the standardized root mean squared residual, the stability of the model using Wald tests, and the coefficient of determination. Means were calculated for the score components of the two indexes with 95% confidence intervals. For the mean estimates, the data were weighted using Iterative Proportional Fitting, applying a basic adjustment for the probability of selection and then fitting marginal proportional totals for age, sex, and area of residence based on the 2011 Estimated Resident Population for Western Australia. Linear regressions on the two components were conducted. Differences at *p* < 0.05 or less were considered to be significant. Stata 13.1 [29] was used for all analyses.

## 3. Results

**Figure 1:**
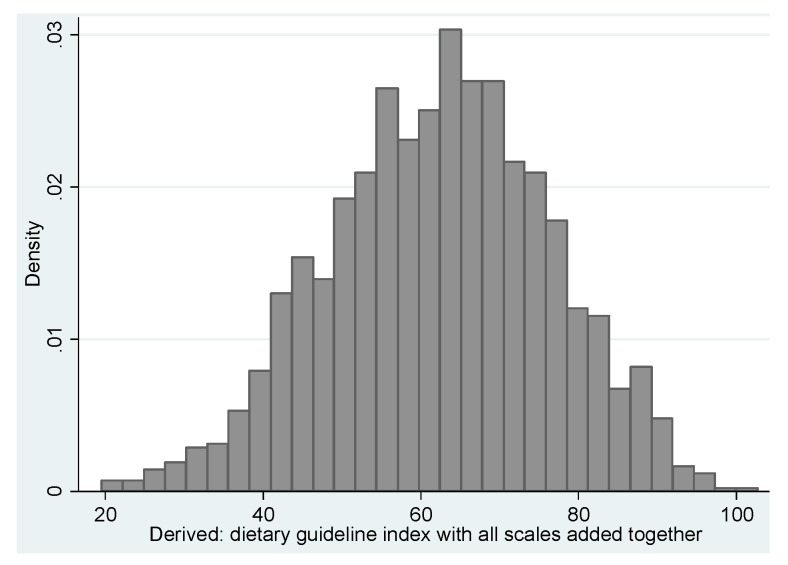
Distribution of the DGI score, NMSS 2012.

The initial NMSS_HEI score showed a wide distribution of scores that has no statistically significant departures from normality for kurtosis but is significantly negatively skewed (Figure 1). The exploratory factor analysis showed two factors, one which reflected the recommended components of the DGI, namely the variety, fruits, vegetables, grains, cereals, dairy, protein, and fluids and one that reflected the discretionary components of the total NMSS_HEI, namely fats, sugar, and additional serves.

**Figure 2:**
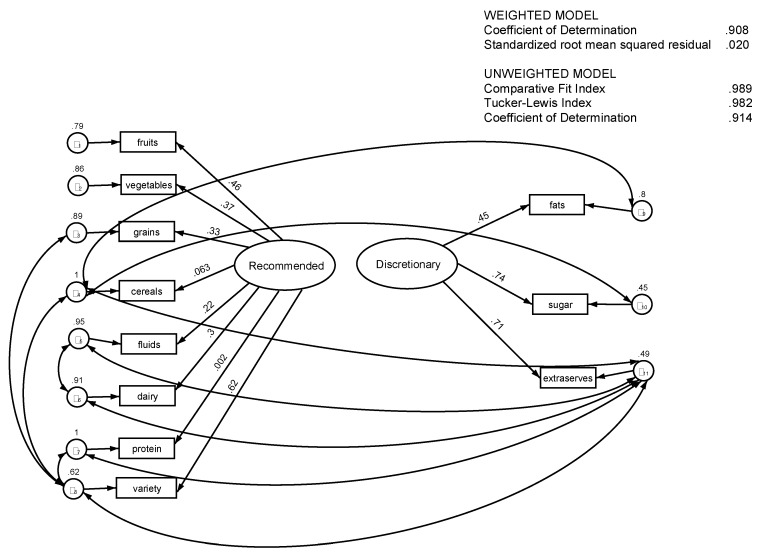
Model produced by structural equation modelling showing two independent components with covariance, NMSS 2012.

The SEM confirmed the two-component structure of the NMSS_HEI and, as with the factor analysis, one reflected the major food groups (Recommended) and the other reflected additional serves and discretionary foods (Discretionary), with each variable contribution to the components statistically significant at *p* < 0.01. Statistically significant covariance were identified for a number of variables using post estimation tests and added to the model with all covariates remaining statistically significant at *p* < 0.05 or better. The addition of the covariance associations altered the p value for the protein score and the cereal score to *p* > 0.05. The largest coefficients (contributors to the model) for the “Recommended” component were variety (β = 0.62, *p* = 0.0001), fruit (β = 0.46, *p* = 0.0001), and vegetables (β = 0.37, *p* = 0.0001), with protein contributing least (β = 0.002, ns). For the “Discretionary” component the contributors were sugar (β = 0.74, *p* = 0.0001), followed by extra serves (β = 0.71, *p* = 0.0001) and fat (β = 0.45, *p* = 0.0001). The model is a non-recursive model and post estimation tests showed it satisfied the stability condition. The raw component scores were negatively correlated but at a very low level (Spearman rho-.078 *p* < 0.05 and in the SEM covariance between the two scores failed to reach statistical significance. For the weighted SEM, the weighted coefficient of determination (CD) was 90.4% and the CD was 91% for the unweighted SEM. The post estimation statistics for the weighted SEM (Table 2) are considered to indicate a good fit with the data [27,30]. For weighted models, no equivalent goodness of fit statistics other than the CD and the standardized root mean squared residual (SRMR) are possible because of the way in which standard errors are estimated, however both the weighted CD and the weighted SRMR are similar to the equivalent measures for the unweighted model. As the data on which the SEM are based are drawn from a cross-sectional population survey, the weighted model coefficients are the most appropriate for use and are the ones displayed in Figure 2.

**Table 2:** Post estimation statistics for the weighted SEM model, NMSS 2012.

Fit Statistic	Value	Description
Likelihood Ratio *		
chi2_ms (33)	51.37	model *vs.* saturated
*p* > chi2	0.02	-
chi2_bs (55)	1749.51	baseline *vs.* saturated
*p* > chi2	0	-
**Population Error**		
RMSEA	0.02	Root mean squared error of approximation
90% CI, lower bound	0.01	-
90% CI, upper bound	0.03	-
pclose	1	Probability RMSEA ≤ 0.05
**Baseline Comparison**		
CFI	0.99	Comparative fit index
TLI	0.98	Tucker-Lewis index
**Size of Residuals**		
SRMR	0.02	Standardized root mean squared residual
CD	0.91	Coefficient of determination

* While the chi square is <0.05, the very large sample size would predict that. The chi square divided by the degrees of freedom is <3 indicating an acceptable chi square for a sample this size [26].

Even though the NMSS_HEI does not capture the whole range of foods eaten, or nutrient intake for the previous day, it does provide a comparable measure at the total component range. Table 3 shows the NMSS_HEI means and proportions meeting the recommended guidelines for the food group. Compared with the DGI_2008, on which the NMSS_HEI is based and which did an assessment of nutrients against the index, many of scales had quite similar means.

**Table 3:** Mean scores for each component identified by the SEM and percentage meeting the recommended dietary guideline in the 2013 ADG by sex with comparisons to the DGI_2008.

	Males			Females		
Dietary Score Component	RFI ^1^	Diff >1 § with DGI_2008	% Meeting RFI ^2^	RFI ^1^	Diff >1 § with DGI_2008	% Meeting RFI ^2^
Food variety	4.96 ± 0.15	-	5.58	5.33 ± 0.10	-	7.00
Vegetables	4.97 ± 0.14	-	8.39	5.66 ± 0.11	-	14.73
Fruit	6.88 ± 0.23	-	58.52	7.74 ± 0.14	-	68.06
Cereals	6.78 ± 0.19	y	38.48	5.98 ± 0.13	-	27.50
Wholemeal/grains	4.64 ± 0.27	y	43.76	4.95 ± 0.19	y	47.35
Protein (meat/fish)	3.54 ± 0.19	y	9.48	3.14 ± 0.13	y	6.79
Dairy	5.00 ± 0.16	-	10.32	4.88 ± 0.12	-	11.37
Fluids ^3^	6.17 ± 0.14	-	15.29	6.11 ± 0.10	y	23.92
**Dietary Score Component**	**DFI ^1^**	**Diff >1 § with DGI_2008**	**% Meeting DFI ^2^**	**DFI ^1^**	**Diff >1 § with DGI_2008**	**% Meeting DFI ^2^**
Fats	7.00 ± 0.14	y	24.49	7.12 ± 0.10	-	29.38
Sugar	6.20 ± 0.2	-	46.07	7.12 ± 0.10	y	58.10
Extra serves	4.01 ± 0.22	-	22.22	4.93 ± 0.17	y	33.83

^1^ Data are mean scores out of 10 weighted using raking; ^2^ Data are percentages meeting recommendations (score of 10) weighted using raking; § The mean score differed by more than 1 when the mean score of the NMSS_HEI was compared to the DGI_2008.

The largest differences were for cereals (mean scale score: DGI_2008 Males 4.2 Females 5.6; NMSS_HEI: Males 6.8 Females 6.0) and eating meats/meat alternatives (mean scale score: DGI_2008: Males 9.8 Females 9.7; NMSS_HEI: Males 3.5 Females 3.1). As the NMSS didn’t ask about consumption of any meat alternatives and as forty percent of the respondents reported that they had not eaten any of the meat or fish, the difference is not unexpected. No obvious explanation exists for the difference in the cereals score unless the DGI_2008 calculation didn’t include breakfast cereals which were included in the NMSS_HEI calculation. It may be that the updated 2013 ADG accounted for some of the differences in the proportions meeting guidelines with increases in the recommended serves of protein, dairy, and cereals in the later version.

Using the two components established by the SEM, a recommended food healthful eating indicator (RF_HEI) and a discretionary food healthful eating indicator (DF_HEI) were calculated by weighting each variable making up the component by the standardised coefficients generated by SEM. Table 4 shows mean scores of selected socio demographic indicators and attitudes. The groups with the highest mean scores for the RF_HEI were people who paid a lot of attention to the health aspects of diet, being retired and doing home duties; the two lowest scores were people who don’t pay any attention to the health aspects of diet and being unemployed. For the DF_HEI the highest mean scores were for people living alone and people who paid a lot of attention to the health aspects of diet; the lowest scores were for people who live in the most socially disadvantaged areas and students.

After controlling for all the variables in table four, *lower* scores for the RF_HEI were significantly associated with lower education levels, having an annual household income less than $40,000, not being able to save any money and paying little or no attention to the health aspects of diet. For the DF_HEI, *lower* scores were significantly associated with being male, not living alone, living in the most socially disadvantaged areas of WA and paying little or no attention to the health aspects of diet.

**Figure 3:**
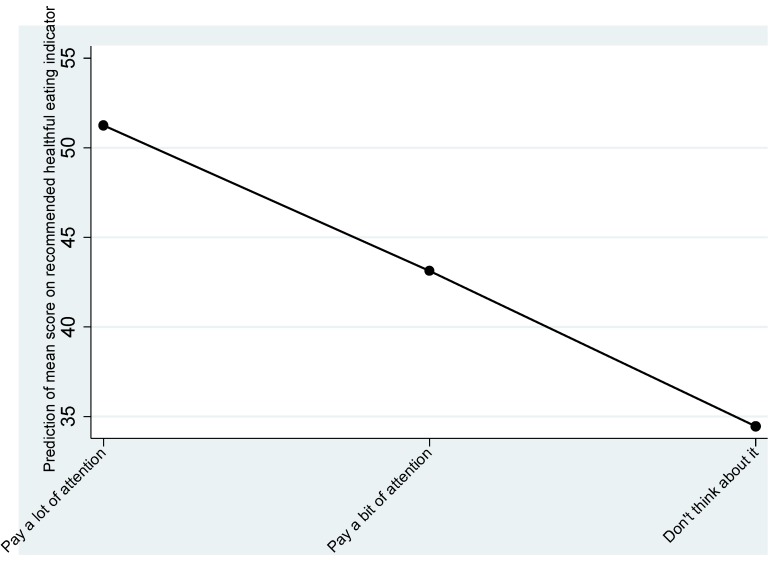
Predictive margins of attention paid to diet.

For the RF_HEI attitudes toward the health aspects of a healthy diet had a linear association with the highest scores associated with paying a lot of attention to diet (Figure 3).

**Table 4:** Mean scores for RF_HEI and DF_HEI by selected socio demographics and attitude toward diet.

Selected Descriptive Variables	RF_HEI	DF_HEI
**Gender**	**Mean (95% CI)**	**Mean (95% CI)**
Male	44.11 (42.50, 45.73)	16.64 (15.77, 17.50)
Female	47.61 (46.46, 48.76)	18.77 (18.10, 19.43)
**Age Group in Years**		
18–44	44.86 (43.30, 46.43)	16.66 (15.82, 17.50)
45–64	47.16 (46.13, 48.20)	17.53 (16.92, 18.14)
**Highest Level of Education Attained**		
Up to Year 12	42.07 (39.50, 44.64)	18.07 (16.67, 19.47)
Year 12	43.40 (40.38, 46.43)	17.00 (15.45, 18.54)
TAFE/Trade	45.98 (44.36, 47.60)	17.89 (17.01, 18.77)
Tertiary	47.89 (46.33, 49.44)	17.70 (16.76, 18.64)
**Annual Household Income**		
Up to $40,000	46.29 (45.26, 47.32)	17.75 (17.16, 18.34)
More than $40,000	41.39 (37.73, 45.05)	17.15 (15.53, 18.78)
**Perceived Discretional Income**		
Can’t save	41.88 (39.69, 44.08)	17.10 (15.96, 18.23)
Can save	47.16 (46.07, 48.26)	17.89 (17.25, 18.53)
SEIFA *	-	-
SEIFA Quintile 1 (most disadvantaged)	43.64 (40.13, 47.15)	14.98 (13.36, 16.59)
SEIFA Quintile 5 (least disadvantaged)	46.96 (45.13, 48.78)	18.25 (17.02, 19.48)
**Current Employment Status**		
Employed	46.35 (45.23, 47.48)	17.94 (17.31, 18.57)
Unemployed	38.28 (31.73, 44.84)	17.78 (13.49, 22.07)
Home Duties	48.32 (46.19, 50.45)	17.28 (15.71, 18.85)
Student	40.85 (36.12, 45.58)	15.66 (13.09, 18.23)
Retired	48.90 (46.38, 51.43)	18.53 (16.88, 20.19)
Unable to work	36.38 (29.35, 43.40)	17.33 (13.23, 21.43)
**Living Arrangements**		
Living with family/partner	45.99 (44.93, 47.04)	17.67 (17.09, 18.25)
Living alone	42.30 (39.24, 45.37)	19.41 (17.82, 21.00)
Other	46.45 (40.25, 52.66)	16.64 (13.02, 20.26)
**Residential Area**	-	-
Metropolitan Perth	45.80 (44.58, 47.02)	17.67 (16.98, 18.36)
Rest of State	46.00 (44.33, 47.67)	17.76 (16.88, 18.64)
**Country of Birth**		
Australia	45.81 (44.11, 47.52)	17.35 (16.43, 18.27)
Other country	45.87 (44.64, 47.11)	17.86 (17.16, 18.56)
**Attention to Health Aspects of Diet**		
Pay a lot of attention	51.47 (50.21, 52.72)	19.23 (18.46, 20.00)
Take a bit of notice	43.17 (41.86, 44.49)	16.68 (15.86, 17.49)
Don’t really think much about it	33.13 (28.93, 37.33)	16.00 (13.98, 18.02)

* Comparison is in that quintile or not.

## 4. Discussion

The aim of this study was to develop a measure that could be used during years when nutrition-based dietary survey data were not available. This proved possible and, while there is no doubt that the RF_HEI and DF_HEI measures do not capture the whole range of foods eaten or have the information to make a nutrient intake assessment, they do provide a basis from which to assess how the population is doing against dietary recommendations. The fact that the initial NMSS_HEI has two independent components offers new information about how the population is approaching their diet. One way is in line with dietary recommendations about serves and types from food groups; the other is in line with dietary recommendations about discretionary foods and additional serves. This means that the same person can have a score indicating healthful eating on one component but not on the other; well on both components or well on neither component. The regression analysis showed that the predictors of eating well for each component are, for the most part, not shared, suggesting that what drives eating behaviours may stem from different influences according to the types of foods being considered. This information is intrinsically different from research, which uses cluster analysis on Australian dietary intake to identify food patterns for example, an eating pattern relatively high in fat and meat compared with an eating pattern higher in fruit and vegetables [31,32], and research using factor, cluster analyses along or ranked regression conducted on data that has not been pre-scored against any standard, such as dietary guidelines [32,33]. These methods identify eating patterns and then explore associations with health indicators [33,34,35], who is eating in line with particular patterns [36,37] and, more recently, other aspects such as how changes in individuals’ dietary patterns affect obesity over time [36,38] and mortality [39]. The two independent components structure identified in this study using SEM suggests that there may be different attitudes and perceptions associated with each that have the potential to inform health promotion and education approaches [14,40]. Population groups such as those with excess weight can now be explored in more detail in relation to their eating choices. The healthful eating indicators as described in this study have not been explored by each of the foods and eating patterns summarised by each indicator. Breakdown of the individual indicators by foods may offer additional information about eating patterns and choices which, in turn, could lead to more precise information about population groups “at risk” due to poor diet. The ability of surveys such as the NMSS to allow the construction of a healthful eating indicator offers a rich source from which to explore important interactions between the psychosocial aspects of diet, such as attitudes, perceptions, and intentions with knowledge and behaviours associated with healthy dietary patterns in the years when detailed nutrient and dietary information with measures of related attitudes and beliefs is not available [41]. The analyses in this paper did not explore interactions or the influence of attitudes on the healthful eating indicators as the aim was to develop healthful eating indicators. To investigate these associations further studies are planned. Investigation of how closely the indicators monitor a more comprehensive measure of consumption, such as a 24 h dietary recall or a three day dietary history, would be valuable to both establish the level of congruence at the scale level and to identify any major gaps.

As with any cross-sectional survey data social desirability may determine some responses but in this case most of the responses are unlikely to be biased in this respect as the respondent would need to be aware of all of the dietary guidelines in formulating their response. In this cross-sectional survey, as in most others, [42] there was an under representation of males relative to females, suggesting a non-response bias for males. The weighting process does adjust for this and having standard errors calculated by robust methods also helps, however, the recommendation for further NMSS data collection is that a stratified random sampling method using area, gender, and age group be considered. Exploration of a more up-to-date source of telephone numbers should also be considered. It is unfortunate that the data from the six surveys could not be pooled but the different data collection methods and different questions for food eaten prohibited this. Consistency in this regard would also be beneficial.

## 5. Conclusions

It is possible to develop healthful eating indicators using validated short dietary questions for use in years when more complete nutrition data is not available. The identification of two independent indicators of healthful eating offers evidence that people approach diet in different ways. This finding suggests that fully investigating each indicator has the potential for better targeted and relevant interventions to improve diet quality in the population.

## References

[1] Belin R.J., Greenland P., Allison M., Martin L., Shikany J.M., Larson J., Tinker L., Howard B.V., Lloyd-Jones D., van Horn L. (2011). Diet quality and the risk of cardiovascular disease: The women’s health initiative (whi). Am. J. Clin. Nutr..

[2] Barker D.J.P. (2004). The developmental origins of adult disease. J. Am. Coll. Nutr..

[3] Barker D., Eriksson J., Forsén T., Osmond C. (2002). Fetal origins of adult disease: Strength of effects and biological basis. Int. J. Epidemiol..

[4] Vaiserman A.M. (2014). Early-life nutritional programming of longevity. J. Dev. Orig. Health Dis..

[5] National Health and Medical Research Council (2013). Australian Dietary Guidelines Incorporating the Australian Guide to Healthy Eating 2013.

[6] Australian Bureau of Statistics (2014). Australian health survey: Nutrition first results—Foods and nutrients, 2011-12. Cat No 4364.0.55.007.

[7] Brambila Macias J., Shankar B., Capacci S., Mazzocchi M., Perez-Cueto F.J.A., Verbeke W., Traill W.B. (2011). Policy interventions to promote healthy eating: A review of what works, what does not, and what is promising. Food Nutr. Bull..

[8] Buttriss J., Stanner S., McKevith B., Nugent A.P., Kelly C., Phillips F., Theobald H.E. (2004). Successful ways to modify food choice: Lessons from the literature. Nutr. Bull..

[9] Baranowski T., Cullen K.W., Baranowski J. (1999). Psychosocial correlates of dietary intake: Advancing dietary intervention. Ann. Rev. Nutr..

[10] Bergea J.M., Wall M., Larson N.J., Forsythd A., Bauere K.W., Neumark-Sztainer D. (2013). Youth dietary intake and weight status: Healthful neighborhood food environments enhance the protective role of supportive family home environments. Health Place.

[11] Larson N., Laska M.N., Story M., Neumark-Sztainer D. (2012). Predictors of fruit and vegetable intake in young adulthood. J. Acad. Nutr. Diet..

[12] Beydoun M.A., Wang Y. (2007). How do socio-economic status, perceived economic barriers and nutritional benefits affect quality of dietary intake among us adults?. Eur. J. Clin. Nutr..

[13] Loth K.A., MacLehose R., Bucchianeri M., Crow S., Neumark-Sztainer D. (2014). Predictors of dieting and disordered eating behaviors from adolescence to young adulthood. J. Adolesc. Health.

[14] Traill W.B., Chambers S.A., Butler L. (2012). Attitudinal and demographic determinants of diet quality and implications for policy targeting. J. Hum. Nutr. Diet..

[15] Pot G.K., Richards M., Prynne C.J., Stephen A.M. (2014). Development of the eating choices index (eci): A four-item index to measure healthiness of diet. Public Health Nutr..

[16] Marks G.C., Webb K., Rutishauser I.H.E., Riley M. (2001). Monitoring Food Habits in the Australian Population Using Short Questions.

[17] Riley M., Rutishauser I.H.E., Webb K. (2001). Comparison of Short Questions with Weighed Dietary Records.

[18] Rutishauser I.H.E., Webb K., Abraham B., Allsopp R. (2001). Comparison of short questions with weighed dietary records. Australian Food and Nutrition Monitoring Unit.

[19] Australian Bureau of Statistics (2013). Socio-Economic Indexes for Areas (Seifa) 2011.

[20] Australian Institute of Health and Welfare (2007). Australian Diet Quality Index Project.

[21] McNaughton S.A., Ball K., Crawford D., Mishra G.D. (2008). An index of diet and eating patterns is a valid measure of diet quality in an australian population. J. Nutr..

[22] National Health and Medical Research Council (2014). Australian guidelines summary dietary. Eat for Health.

[23] Oyebode O., Gordon-Dseagu V., Walker A., Mindell J.S. (2014). Fruit and vegetable consumption and all-cause, cancer and cvd mortality: Analysis of health survey for England data. J. Epidemiol. Community Health.

[24] National Health and Medical Research Council (2013). Eat for health. Educators Guide.

[25] Imamura F., Jacques P.F. (2011). Invited commentary: Dietary pattern analysis. Am. J. Epidemiol..

[26] Schreiber J.B. (2008). Core reporting practices in structural equation modeling. Res. Soc. Adm. Pharm.

[27] Iacobucci D. (2009). Everything you always wanted to know about sem (structural equations modeling) but were afraid to ask. J. Consum. Psychol..

[28] Muthen B.O., Satorra A. (1995). Complex sample data in structural equation modeling. Sociol. Methodol..

[29] StataCorp (2013). Stata glossary and index release 13^®^. Statistical Software.

[30] Hu F.B., Bentler P.M. (1999). Cut off criteria for fit indexes in covariant structure analysis: Conventional criteria *versus* new alternative. Struct. Equ. Model..

[31] Grieger J.A., Scott J., Cobiac L. (2012). Cluster analysis and food group consumption in a national sample of Australian girls. J. Hum. Nutr. Diet..

[32] Moeller S.M., Reedy J., Millen A.E., Dixon L.B., Newby P.K., Tucker K.L., Krebs-Smith S.M., Guenther P.M. (2007). Dietary patterns: Challenges and opportunities in dietary patterns research: An experimental biology workshop, 1 April 2006. J. Am. Diet. Assoc..

[33] Li W.-Q., Park Y., Wu J.W., Goldstein A.M., Taylor P.R., Hollenbeck A.R., Freedman N.D., Abnet C.C. (2014). Index-based dietary patterns and risk of head and neck cancer in a large prospective study. Am. J. Clin. Nutr..

[34] Amini M., Shafaeizadeh S., Zare M., Boroujeni H.K., Esmaillzadeh A. (2012). A cross-sectional study on food patterns and adiposity among individuals with abnormal glucose homeostasis. Arch. Iran. Med..

[35] Xu B., Houston D., Locher J.L., Zizza C. (2012). The association between healthy eating index-2005 scores and disability among older Americans. Age Ageing.

[36] Elstgeest L.E.M., Mishra G.D., Dobson A.J. (2012). Transitions in living arrangements are associated with changes in dietary patterns in young women. J. Nutr..

[37] Kant A.K. (2004). Dietary patterns and health outcomes. J. Am. Diet. Assoc..

[38] Pachucki M.A. (2012). Food pattern analysis over time: Unhealthful eating trajectories predict obesity. Int. J. Obes..

[39] Kant A.K., Schatzkin A., Graubard B.I., Schairer C. (2000). A prospective study of diet quality and mortality in women. J. Am. Med. Assoc..

[40] Lê J., Dallongeville J., Wagner A., Arveiler D., Haas B., Cottel D., Simon C., Dauchet L. (2013). Attitudes toward healthy eating: A mediator of the educational level-diet relationship. Eur. J. Clin. Nutr..

[41] Grunert K.G., Shepherd R., Traill W.B., Wold B. (2012). Food choice, energy balance and its determinants: Views of human behaviour in economics and psychology. Trends Food Sci. Technol..

[42] Galea S., Tracy M. (2007). Participation rates in epidemiologic studies. Ann. Epidemiol..

